# Robots in Nursing Homes: Helping Nurses Detect and Prevent Falls

**DOI:** 10.20900/agmr20250001

**Published:** 2025-01-03

**Authors:** Yuval Malinsky, Lynn McNicoll, Stefan Gravenstein

**Affiliations:** 1 Vigorous Mind, Inc., Natick, MA 01760, USA; 2 Department of Medicine, Alpert Medical School of Brown University, Providence, RI 02903, USA; 3 Brown University Health (Formerly Lifespan), Providence, RI, 02903, USA; 4 Providence Veterans Administration Hospital, Providence, RI 02908, USA

**Keywords:** fall detection, fall prevention, robot, nursing homes, long lying

## Abstract

Falls are a leading cause of morbidity and mortality in older adults, especially among nursing home residents. Falls occur more commonly among older adults with dementia than among those without dementia. Moreover, half of nursing home residents have moderate to severe cognitive impairment. While less than 5% of older adults live in nursing homes, they account for 20% of deaths from falls in this age group. In addition, 78% of older adults who fall need help in getting up from the floor. The consequences of falling, such as prolonged lying on the floor, can produce severe and prolonged health effects. The acute shortage of staff in nursing homes, especially during evening, night and weekend shifts, can delay the detection and response to falls. There are various systems designed to detect falls and alert staff, including those utilizing wearable devices, ambience sensors and cameras (vision) as well as fusion systems. They each have their advantages and drawbacks. In this NIA-funded SBIR grant, we are developing and testing the feasibility of a fall detection and prevention system that addresses the drawbacks of previous systems. We anchor our approach on the deployment of an autonomously navigating robot equipped with a mounted infrared camera and machine learning software designed to detect the risk of falls and falls themselves. The robot will patrol resident rooms during evening and night shifts and alert the staff, allowing them to evaluate the fall risk or fall alert presented by the robot video camera and determine whether indeed a resident has fallen or is at risk of falling, and take appropriate action.

## INTRODUCTION

In 2018, 36.5 million falls occurred in older adults in the USA, with 8.4 million resulting in injuries [1]. The death rate from falls among persons aged ≥65 years increased by 31% between 2007 and 2016, with persons aged ≥85 years experiencing the fastest growing death rate [2]. Each year, between half and three-quarters of nursing home residents fall, with some residents falling multiple times, resulting in a mean fall rate of 1.7 falls per person-year [3,4]. While less than 5% of adults 65 and older live in nursing homes, these residents account for 20% of deaths from falls in this age group. Half of nursing home residents experience moderate to severe cognitive impairment [5]. Older adults with Alzheimer’s disease and related dementias (AD/ADRD) fall more frequently than their cognitively intact peers and suffer more serious injuries (e.g., hip fractures) and adverse consequences [6,7].

All too often, falls lead to serious consequences, especially for frail older residents. Residents who fall without injury may develop a fear of falling, leading them to self-impose activity limitations. This results in progressive weakness (which may further increase fall risk), decreased ability to function, depression, social isolation and reduced quality of life [4,8]. Fall-related injuries reduce normal functioning ability and triple the risk of emergency department visits or hospitalizations. Most falls (66%–75%) occur in the resident’s room, with almost half (48%) resulting in an injury [3,9]. One in every 10 residents who fall sustains a serious injury. Falls peak between 2 p.m. and 8 p.m. [3,9], and evening falls are more likely to result in serious injuries than daytime falls.

In addition, 78% of individuals aged 65 and older who fall require assistance to get up from the floor [10]. This inability to rise from the ground after a fall poses a further danger to the individual due to the extended time spent lying on the floor [11]. While lying on the ground, individuals may experience serious physical and psychological effects. Researchers have reported abnormal laboratory parameters, dehydration, rhabdomyolysis, sepsis, infections, pressure, and loss of consciousness in such cases [10].

The COVID-19 pandemic exacerbated pre-existing nursing home staff shortages. During the first two years of the pandemic, nursing homes lost 241,000 employees—15.2% of their workforce [12]. As of March 2024, prior workforce levels have not recovered; for 72% of nursing homes, staffing remains lower than pre-pandemic levels. Active recruitment for nurses’ aides is ongoing in 95% of nursing homes, nurses in 89%, and licensed practical nurses in 85% [13]. The increased burden on the existing nursing homes’ staff and the ongoing shortage of staff, especially during evening, night and weekend shifts, increases the risk that staff do not detect falls as well as respond slowly to falls when they do occur.

Researchers introduced technologies to address fall detection in the early 2000s. Most fall detection methods fall into three broad categories: wearable device-based, ambience sensor-based and camera (vision)-based systems [14–16]. Recently, researchers have introduced fusion and personalized-adaptive threshold-based detection systems. Wearable device-based detection systems use sensors such as accelerometers and gyroscopes that users wear on their bodies to measure motion. Manufacturers typically produce these as bracelets, bands, smart clothing or smartphone applications. Wearable device-based systems offer advantages such as low price and easy setup. However, they have disadvantages: users may find them intrusive, nursing home residents may not be able to use them, recharging can be challenging and their robustness varies. Ambience sensor-based detection systems incorporate sensors into the environment rather than having residents carry them. These include pressure sensors, acoustic sensors on the floor, infrared array sensors, radiofrequency (RF) and radar-based systems mounted on walls and WiFi signal-based detection. While these systems do not intrude on users, they typically cost more, require longer setup times and need more maintenance than wearables. In some cases, ambience sensor-based systems lack specificity, which produces false-positive alarms and alarm fatigue. For example, some recent studies suggest that pressure sensor alarms alone do not reduce falls, contributing to many nursing homes discontinuing their use [16–18]. Camera (vision)-based detection solutions do not require sensors. However, they have drawbacks such as blind spots, distance limitations from the camera (especially when using Kinect), sensitivity to illumination changes and privacy concerns. Fusion-based solutions combine different detection methods to increase reliability, but this also increases their complexity.

In this one-year Phase I SBIR grant, we propose a solution which uses an infrared video camera mounted on an autonomously navigating robot to patrol rooms of nursing homes (where doors are always kept open). Using machine learning, the system will detect falls of residents who are trying to get out of bed and then alert staff. We address the issue of blind spots that static cameras have by using a robot that can move into different spots in the room to detect falls. The infrared night vision video camera allows us to address both the illumination challenge and privacy. In addition, when the robot alerts staff at the nursing station, staff can utilize the robot’s video camera view to see whether a resident has fallen or is about to get out of bed, and they can communicate with the resident through the robot’s audio system. This minimizes false alarms and alarm fatigue and can calm residents knowing that help is on the way. The robot will be deployed in three dementia units of nursing homes and will patrol the rooms of 110 residents for three months.

We hypothesize that we will achieve significant improvement in specificity for correctly identifying falls and largely eliminate fall-like events being misidentified as falls while shortening the time to fall detection and response. In addition, fall prevention could result from the robot’s interaction with the residents, where it will speak to residents who are attempting to get out of bed or a chair and ask them to wait for help.

The goal of this project is to demonstrate the feasibility of using an autonomously navigating robot with an infrared camera and machine learning algorithms for detecting falls and fall risks and alerting nursing home staff (Vigorous Mind Robot—“VMR”). If Phase I is successful in demonstrating the feasibility, we plan to submit a three-year Phase II grant application where the robot will be deployed in thirty nursing homes units with a total of 1000 residents for six months, with the goal of proving that using the robot significantly shortens the response time after falls, reduces falls and hospitalizations due to falls in nursing homes and improves resident and staff satisfaction.

## AIMS OF THE PROJECT

### Aim 1: Development

During the development, we will do the following: (1) We will integrate an infra-red camera into the autonomously navigating robot as well as image processing and machine learning software that applies posture analysis to detect if a person fell or is trying to get out of bed. (2) We will train the machine learning algorithms using a library of fall images that we acquire elsewhere or using new images that we take ourselves. The machine learning model will be trained on a diverse dataset of human postures, including individuals on the floor, sitting down, standing and lying on beds, each captured in various positions. The dataset will include images taken during both daytime and nighttime using a night vision camera. These images will be organized into three categories: fall detection (people on the floor), fall prevention (people sitting or standing) and bed monitoring (people lying on beds). (3) We will program the robot to turn on its video camera and alert staff when it identifies a fall or a fall-risk behavior, as well as turn on ambient light if this occurs at night. The alert will be a call to an application on a tablet where a map shows the location of the robot. When staff pick up the call, they will be connected to the robot’s video camera. This will allow staff to see if a resident has fallen or is at risk of falling and to communicate with the resident through the robot video camera, assess the situation and take the required action.

### Aim 2: Usability Testing

Here, our goal is to see that the system performs the functions according to its design parameters and that both staff and residents feel comfortable with it. We will test the following: (1) First, we will test whether the robot can detect falls and residents trying to get out of bed during the day and at night. (2) Next, we will test whether the robot will say the following when a resident has fallen: “This is Temi the robot. I am getting help right away”. Similarly, if a resident is known as prone to falling (“frequent faller”) and is trying to get out of bed, then we will test whether the robot says the following: “This is Temi the robot, please sit down. I am calling for help”. (3) Finally, we will test whether the alert system of the robot works, including turning on an ambient light and allowing staff to connect to the robot’s video camera, see the resident and assess the situation from the nursing station. Tests will be conducted initially at the company’s demo nursing room and then at the nursing home with Vigorous Mind staff imitating falls and fall risks. Then, the robot will begin patrols for a limited number of residents’ rooms under the supervision of a research assistant with company staff available at the building to fix any technical issues uncovered.

### Aim 3: Feasibility Study of VMR for Detecting and Preventing Falls in Nursing Homes

We will conduct a feasibility pilot study of deploying the VMR in three dementia units in two nursing homes in Rhode Island, for three months. We will program the robots to visit the rooms of “frequent fallers” more frequently and visit all rooms on a regular basis and “peek” to see that no resident has fallen or is about to get out of bed. The infrared camera, which facilitates night vision, scans the room from several preset locations to ensure that it scans the entire room. If the robot detects a fall or a resident attempting to get out of bed, it will alert the staff as described above. Once staff has terminated the communication with the resident through the robot or the event is determined to be a false alarm, the robot will either be instructed by staff or follow a pre-programmed automation to continue the patrol or another task from the point it was interrupted. Based on initial tests, we expect that the rooms of “frequent fallers” will be visited 2–3 times per hour and regular rooms 1–2 times per hour. The robot battery can last for eight hours.

## STUDY DESIGN AND DATA COLLECTION

There are a total of 110 residents in the units of the participating nursing homes. The units have both private rooms and semi-private rooms that the robot will patrol and scan for falls and fall risk. Inclusion and exclusion criteria for participating in the feasibility study are shown in Table 1.

The robots will be deployed in the units about one month before starting the usability testing and will be programmed to play music in the corridors three times per day so the residents get used to the robots and associate them with something fun and pleasing. This has been successfully tested by us in multiple nursing homes’ dementia units in the last two years. Research staff will have the list of residents and assign each resident a subject number. Subject numbers of residents, their room number and risk of falling measure are kept in VMR. VMR will automatically build its fall detection round, visiting the “frequent fallers” rooms twice during each round. When new residents are admitted, subject number, room number and their fall risk will be entered into the VMR system, and the robot will adjust the rooms where it visits more frequently accordingly. In the future, this update will be automated with an interface with the nursing home electronic health records system (EHR). The robot will conduct the rounds during the evening and night shifts as defined by staff. Staff will have a tablet at the nurse station where a live map showing the robot’s location will be presented. When the robot detects a fall or a risk of fall, the tablet will ring (similar to a phone ring) and show a button that staff can click on to connect to the robot’s video camera. Through the video camera, staff will be able to see if a resident has fallen or is trying to get out of bed and will assess the situation and communicate with the resident. Staff will be able to instruct the robot to continue the patrol if there is no fall or they are handling the situation. If the robot detects that there is more than one person in the room and no one on the floor, it will leave the room. If there is another person in the room and another on the floor, it will alert staff. If the second person in the room is a staff member, s/he will be able to click a button on the robot screen to instruct it to continue the patrol. If it is a semi-private room that is home to two residents, the robot will check on one resident, and if s/he is not at risk, it will turn around and check the status of the second resident. If both are resting, the robot will leave the room without interrupting the residents. If it detects that a resident is on the floor, it will perform the procedure described above. If a staff member is already in a semi-private room where there was a fall, s/he will be able to send the robot to continue the patrol or task as described above for the private room. All fall alerts and details of the communication with the resident (including start time and end time) will be logged by VMR for future analysis. Falls reported by the staff and robot will be tabulated according to their concordance: (1) the robot correctly detected and reported a fall but the staff did not; (2) the staff reported a fall but the robot did not; or (3) the robot correctly detected and reported a fall and the staff also reported a fall.

Our primary outcome measure will be the percentage of time that the robot correctly detected falls to the ground and alerted staff. Secondary outcomes will be as follows: (1) the percentage of time that the robot successfully turned on the ambient light and the video camera and communication with the resident worked; (2) the percentage of staff that felt that the robot improved their ability to provide safe care; (3) the percentage of time that the robot detected that a resident is trying to get out of bed and alerted the staff; (4) the percentage of time that the robot was the first responder to a fall situation; and (5) the percentage of false-positive alerts. Table 2 lists the outcome measures.

Successful completion of this Phase I project will prepare us for a much larger population Phase II project as described above to prove that using the robot significantly shortens the response time after falls, reduces falls and subsequent hospitalizations in nursing homes and improves resident and staff satisfaction. The long-term goal is to establish that early fall detection may reduce morbidity and mortality associated with falls.

## Figures and Tables

**Table 1.** Inclusion and exclusion criteria.

**Inclusion Criteria**

• Nursing home residents living in the dementia memory unit or a long-term care unit

**Exclusion Criteria**

• Residents on isolation precautions (e.g., *Clostridoides difficile,* COVID-19, MRSA)
• Actively dying resident
• Resident and/or family decline
• Functional or structural quadriplegia with inability to mobilize with little to no risk of falling
• Residents who become agitated when the robot engages with them

**Table 2.** Outcome measures.

Feasibility Outcomes	Collection Method	Definition	Success Criteria

% Success	VMR system and staff	Robot correctly detected that a resident fell and alerted staff (Primary Endpoint)	≥95% of the time
% Success	VMR system and staff	The robot ambient light, video camera and communication with the resident systems worked	≥95% of the time
% Agree	Staff interview	The fall detection system improves our ability to provide safe care	≥75% agree or strongly agree
% Success	VMR system and staff	Robot detected correctly that the resident is trying to get out of bed and alerted staff	≥85% of cases where the IR sensor alerted the robot
% Success	VMR system and staff	Robot detected fall before staff	>30% of falls detected
% of false positives	VMR system	Times robot alerted the staff falsely of a fall or a risk of fall out of the total number of alerts	≤10% of alerts

## Data Availability

No data were generated from the study.
